# Supplementary material for: Content-rich biological network constructed by mining PubMed abstracts
Source: BMC Bioinformatics. 2004 Oct 8;5:147. doi: 10.1186/1471-2105-5-147 (PMC528731; doi:10.1186/1471-2105-5-147)
Supplement: Additional File 2 — The original results of the above study (non-essential files are deleted to keep the file size under the limit set by BMC bioinformatics). [file 1471-2105-5-147-S2.bz2 › chilibotAdditionalFile2/dip20/440ID9153236E10391/html/CCR7_CCL19.html]

 


 **CCR7** and **CCL19** 
  
Found 79 abstracts in PubMed, retrieved 20.  
 

 What does Google say? 
 PDF only 
| .edu only 

---

**Interactive relationship** (e.g. stimulation, inhibition, etc)

**Neutral relationship**- **CCR7**  binds  **CCL19**  and CCL21, causing the highly activated DCs to be trapped in the peripheral tissue site.  Ref: 12414528 Am J Pathol, 2002
- In contrast, the majority of DC Lamp mature DCs in inflamed skin were totally negative for  **CCL19**  and were surrounded by  **CCR7**  memory type T cells.  Ref: 12474232 J Pathol, 2003
- Using immunohistochemistry, the majority of DC Lamp mature DCs in the T cell area of LNs expressed  **CCL19**  and were surrounded by  **CCR7**  naïve type lymphocytes, while CCL21 was expressed in reticular stromal cells and vascular endothelial cells.  Ref: 12474232 J Pathol, 2003

**Non-interactive relationship** (e.g. studied together, co-existance, homology, etc.)

- We studied the role of Rho kinase and extracellular signal regulated kinase ERK 2 in the polarization and migration of T lymphocytes in response to the  **CCR7**  ligands EBI1  [ **CCR7** ]  ligand chemokine ELC  [ **CCL19** ] .  Ref: 12729902 FEBS Lett, 2003
- In normal skin, on the other hand, no cells immunoreactive for  **CCL19** , CCL21, or  **CCR7**  were found.  Ref: 12474232 J Pathol, 2003
- Within 24h of local antigen priming, the expression of three chemokine receptor genes CCR3, CCR5, and  **CCR7**  and three chemokine ligand genes CCL12,  **CCL19** , and CCL25 were significantly upregulated.  Ref: 12705880 Biochem Biophys Res Commun, 2003
- The CC chemokine receptor CCR 7  [ **CCR7** ]  ligands CCL21 and  **CCL19**  were recently described as essential elements for establishing the microenvironment needed to initiate optimal immune responses in secondary lymphoid tissues.  Ref: 12890630 Virology, 2003
- The binding to IP 10 could be inhibited by SLC but not by a non mac25 AGM binding chemokine, EBI1  [ **CCR7** ]  ligand chemokine ELC  [ **CCL19** ] .  Ref: 12847218 J Immunol, 2003
- Mesenchymal cells also secrete  **CCL19** , a ligand for  **CCR7** , yet it is not clear whether  **CCR7**  and  **CCL19**  are important for secondary lymphoid organ development.  Ref: 12732661 J Exp Med, 2003
- In the present study, we examined the effects of  **CCL19**  and CCL21, two  **CCR7**  ligands, on endocytosis of fluorescein isothiocyanate FITC dextran by murine DCs.  Ref: 12609829 Blood< MedlineTA>Blood, 2003
- The U12 gene encoded a calcium mobilizing receptor for the EBI1  [ **CCR7** ]  ligand chemokine macrophage inflammatory protein 3beta ELC  [ **CCL19** ]  MIP 3beta but not for other chemokines, suggesting that the chemokine selectivity of the U12 gene product is distinct from that of the known mammalian chemokine receptors.  Ref: 12829849 J Virol< MedlineTA>J Virol, 2003
- RT PCR analysis revealed expression of  **CCL19** , CCL21, and  **CCR7**  at high levels in LNs and at low levels in inflamed skin.  Ref: 12474232 J Pathol, 2003
- The lymphocyte expressed chemokine receptor  **CCR7**  is playing an important role during this process, as its HEV presented ligands  **CCL19**  and CCL21 can trigger rapid integrin activation under flow in addition to inducing a chemotactic response, which may participate in transmigration and or interstitial migration.  Ref: 12393730 Blood, 2003
- SIV infection altered the expression of chemokines  **CCL19**  MIP 3beta, CCL21 6Ckine, and CCL20 MIP 3alpha and of chemokine receptors  **CCR7**  and CCR6 that drive DC trafficking.  Ref: 12406887 Blood, 2003
- Interestingly, a second  **CCR7**  ligand,  **CCL19** , triggered T cell sticking in cremaster muscle venules, but failed to induce extravasation in air pouches.  Ref: 12707342 J Immunol, 2003
- **CCL19**  MIP 3beta, CCL20 MIP 3alpha, CCR6, and  **CCR7**  expression increased in lymph nodes during the early systemic burst of viral replication acute infection.  Ref: 12406887 Blood, 2003
- To analyse the nature of T cell mature DC interactions further in chronically inflamed skin and LNs, the present study focuses on another chemokine system, namely  **CCL19**  EBI1  [ **CCR7** ]  ligand chemokine.  Ref: 12474232 J Pathol, 2003
- Transcripts for  **CCL19**  and CCL21 and their common receptor  **CCR7**  were induced in the CNS of mice undergoing relapsing remitting and chronic relapsing EAE.  Ref: 12580544 Brain Pathol, 2003
